# Successful outcomes of pediatric dacryocystorhinostomy with adjunctive 5-fluorouracil

**DOI:** 10.3389/fopht.2026.1805226

**Published:** 2026-07-17

**Authors:** Alina K. Sinha, Ethan L. Kahana, Tomas E. Meijome, Adam Jacobson, Jane Z. Spadaro, Stella Y. Chung, Alon Kahana

**Affiliations:** 1 Department of Ophthalmology, Oakland University William Beaumont School of Medicine, Royal Oak, MI, United States; 2 College of Literature, Science and Arts, University of Michigan, Ann Arbor, MI, United States; 3 Indiana University Health Ophthalmology, Indianapolis, IN, United States; 4 Department of Ophthalmology and Visual Science, Kellogg Eye Center, University of Michigan, Ann Arbor, MI, United States; 5 Kahana Oculoplastic and Orbital Surgery, Livonia, MI, United States; 6 Department of Ophthalmology, New York University Grossman School of Medicine, New York, NY, United States

**Keywords:** 5-fluorouracil, 5-FU, dacryocystorhinostomy, DCR, epiphora, fluorouracil, nasolacrimal duct obstruction, pediatric

## Abstract

**Purpose:**

To determine whether nasal intramucosal 5-fluorouracil (5-FU) injection improves outcomes after external dacryocystorhinostomy (DCR) in pediatric patients with nasolacrimal duct obstruction (NLDO).

**Methods:**

Multi-center retrospective non-comparative case series review of all DCR cases with intra-operative 5- FU injection performed between 2014 and 2023 on all patients age < 18 years. Each case was reviewed to determine if the patients had epiphora recurrence following surgery with 5-FU injection.

**Results:**

Sixteen pediatric patients with NLDO (19 eyes) underwent DCR and nasal intramucosal injections of 5-FU intra-operatively. One had a lost stent. Ten (53%) eyes were in patients with craniofacial syndromes. In all patients with the use of 5-FU there was no recurrence of tearing. Eight of the patients (42%) had already had at least one or two failed stents prior in which recurrence had occurred. There were no post-operative complications.

**Conclusion:**

Adjuvant intramucosal 5-FU demonstrated a 100% success rate of epiphora not recurring following pediatric DCR without any complications in this small sample size, particularly in patients with increased risk of recurrence.

## Introduction

Most cases of pediatric nasolacrimal duct obstruction (NLDO) are due to congenitally incomplete canalization of the nasolacrimal duct (1, 2). Obstruction of the nasolacrimal duct can cause excessive tearing and possibly dacryocystitis. The reported incidence of pediatric NLDO ranges from 5% to 20% (2, 3). Though most cases of NLDO resolve with observation and lacrimal sac massage, persistent cases may require surgical intervention (4).

First line surgical treatment of congenital NLDO is nasolacrimal duct probing, which is successful in approximately 80% of cases (5). Treatment options after failed lacrimal duct probing include repeat probing, repeat probing with temporary lacrimal system stenting, balloon catheter dacryoplasty, and surgical dacryocystorhinostomy (DCR) (6–9). Outcomes after external or endoscopic DCR in pediatric patients with congenital and acquired NLDO range from 83 to 97% (10–18). Though DCR is safe and effective in treating most pediatric patients with NLDO, patients with associated craniofacial abnormalities have poor outcomes after DCR and remain difficult to treat (19).

The challenge of pediatric DCR is 3-fold: the anatomy is small and crowded, children have robust inflammatory response that can lead to scarring over the osteotomy site, and children’s bones are highly osteoblastic, resulting in healing of the osteotomy to narrow and even close it. Anti-metabolites have been used as part of various surgeries in order to decrease the fibrotic response by inhibiting post-operative fibroblast proliferation and function. Mitomycin C (MMC) and 5-fluorouracil (5-FU) are two pharmacologic agents that have been shown to be safe adjuncts in glaucoma filtering surgery (20–24). MMC and 5-FU have been used to inhibit scarring and cicatrix formation over the osteotomy site in DCR, though evidence has been mixed regarding improvement in outcomes (25–37). MMC is more commonly used, usually delivered on a pledget placed on the mucosa for a brief period of time (2–15 minutes) (34). However, MMC is an alkylating agent with significant risk of collateral damage to surrounding tissue and a narrow therapeutic window (38, 39). To our knowledge, no prior studies have examined the role of intraoperative nasal intramucosal 5-FU injections on DCR outcomes in the pediatric population. In this study, we perform a retrospective analysis to determine whether adjuvant 5-FU can improve success rates following pediatric DCR without significant complications.

## Materials and methods

### Study design

This research study was HIPAA compliant and adhered to the ethical principles outlined in the Declaration of Helsinki as amended in 2013. IRB approval was obtained at both University of Michigan and William Beaumont Hospital (40783 and 2021-126, respectively). We performed a retrospective non-comparative case series review for patients 18 years of age and younger who underwent DCR with adjunctive 5-FU by multiple oculoplastic surgeons at the University of Michigan and Kahana Oculoplastic and Orbital Surgery from January 1, 2014, to August 1, 2023. Patients were identified searching the electronic health record for CPT code 68720. Three patients were excluded due only a probing was performed and one patient was lost to follow-up. Each chart was reviewed for demographic information, use of intramucosal 5-FU, presence of a craniofacial disorder or surgical comorbidity, post-operative complications, and post-operative recurrence. The surgeon’s decision to use 5-FU was determined by if cases were higher risk for surgical failure. Surgical comorbidity was defined as any non-craniofacial anatomical abnormality which could potentially affect the outcome of a DCR, such as history of trauma to the lacrimal system, lacrimal tumors or congenital facial abnormalities. Recurrence was defined as persistent symptoms of tearing or epiphora as well as analysis of tear lake under slit lamp exam at time of post-operative follow up. Each eye was treated independently in the dataset.

### Surgical procedure

The incision was made at the nasojugal fold followed by sharp dissection down to the anterior lacrimal crest with a monopolar unit and microdissection needle. The periosteum was incised and elevated with a Freer to expose bone. The lacrimal bone was fractured, and then Kerrison rongeurs were used to create a large osteotomy. The osteotomy was extended superiorly to the superior edge of the lacrimal sac, to include bone that would be immediately across from the internal punctum. In older children, the anterior ethmoid air cells were identified and carefully removed with Takahashi forceps. An anteriorly-based nasal mucosal flap was created, creating an opening into the nasal passages. After placing the lacrimal stent through the canaliculi, the internal punctum was carefully visualized and treated to avoid any loose mucosa around the stents. The lacrimal and nasal mucosa around the ostium were infiltrated with between 0.5 ml and 3ml of 50mg/ml 5-FU submucosally depending on age, size and area of injection. The mucosal flaps were sutured together to cover the ostium.

## Results

Nineteen eyes of 16 patients and their demographic data were identified (**Table 1**). The average age was 5.18 years (median 3.18 years, range 1–18 years). All patients had previously failed probing and irrigation and 3 had one failed stent placement (16%), 4 had two failed stent placements (21%), and 1 had a balloon dacryoplasty (5%). In one eye the stent was lost following the DCR procedure with 5-FU injection (5%). Craniofacial syndrome was present in 10 of 19 eyes (53%), including 5 eyes (3 patients) with Down Syndrome, 2 eyes (1 patient) with Arid Syndrome, 1 eye with Apert Syndrome, 1 eye with Brachio-oculo-facial syndrome, and Rubenstein-Taybi Syndrome. Mean length of follow up was 2.36 years. No intraoperative or postoperative complications were encountered. Recurrence of epiphora following DCR with adjunctive nasal intramucosal injection occurred in 0 of 19 eyes.

**Table 1:** Table of demographic data of all patients who underwent DCR with adjunctive 5-FU and were included in the study.

Patient demographics and surgical characteristics	DCR with 5-FU n, (%)
Total number of eyes*	19
Sex	
Male	13 (68%)
Female	6 (32%)
Eye	
Right	9 (47%)
Left	10 (53%)
Procedure Type	
External	19 (100%)
Surgical Comorbidity	
Lost stent	1 (5%)
Craniofacial Disorder	
Apert Syndrome	1 (5%)
ARID1B Syndrome	2 (11%)
Branchio-Oculo-Facial Syndrome	1 (5%)
Down Syndrome	5 (26%)
Rubinstein-Taybi Syndrome	1 (5%)

DCR, dacryocystorhinostomy; 5-FU, 5-flurouracil.

*All categories are based off eyes that underwent DCR not patients

(as 3 males underwent bilateral DCR)

## Discussion

In this study, we show that adjunct intramucosal 5-FU for pediatric DCR was associated with a 100% resolution of epiphora. Our findings are comparable to prior published literature of DCR in pediatric patients, ranging from 83 to 97% (10–17).

Congenital NLDO is common, and a minority of patients requires reconstructive surgery with bypass of the non-functional duct through DCR. Pediatric patients represent a unique challenge because the anatomy is small and crowded, they have a robust inflammatory response, and the surgical osteotomy is prone to narrowing secondary to increased osteoblastic activity. Pediatric patients can also present with surgical comorbidities from prior procedures or related to congenital craniofacial disorders. In this study, we found that intramucosal 5-FU in pediatric patients undergoing DCR for NLDO is associated with no epiphora recurrence, without any reported complications in this small sample size.

Antimetabolites have been adopted in ophthalmology for a wide range of indications. 5-FU and MMC have been shown to be safe adjuncts in glaucoma filtering surgery by reducing conjunctival scarring (20–24). Following exposure, 5-FU or MMC are rapidly transported into cells where they elicit their effects on DNA replication and cellular physiology (40, 41). Topical application of MMC during trabeculectomy decreases risk of bleb failure and improves long term control of intraocular pressure (22, 23). Other indications for ocular or periocular MMC and 5-FU include primary and secondary treatment of ocular surface squamous neoplasia, prevention of recurrence of pterygium after surgical excision, and in cicatrizing conjunctival disorders to reduce vision threatening complications (42–45). Topical MMC applied to nasal mucosa on a pledget has also been used as a safe adjunct to decrease fibrosis in endoscopic sinus surgery and in repair of choanal atresia in children (46, 47). MMC is a potent alkylating antimetabolite, and use during glaucoma and pterygium surgeries has been associated with increased risk of tissue necrosis, trabeculectomy bleb infection, and endophthalmitis (48–50). Hence, adjunct use of MMC in ophthalmic surgeries has been limited to high-risk cases. Use of 5-FU in glaucoma surgery has been associated with increased risk of epitheliopathy and hyphema, but these adverse events were not found to be statistically significant in a large metanalysis (22).

Failure of DCR can be caused by surgical technique, e.g. inadequate osteotomy size or location, nasal cavity abnormalities, or post operative healing that results in narrowing of the osteotomy or obstruction of the intranasal osteotomy site by granulation or scar tissue (10, 19). Antimetabolites have been used safely as adjunct to DCR to decrease post-operative fibrosis, though evidence for improved outcomes has been mixed (25–37). The majority of studies of adjunct antimetabolite during DCR have focused on use of topical MMC in adult patients. A recent meta-analysis of 31 studies suggests improved functional and anatomical outcomes after DCR with topical intraoperative MMC at follow up time points at least 6 months from surgery. However, the effect was modest with a relative risk of 1.12 and 1.25 for functional and anatomic success respectively (27). No significant adverse effects were reported secondary to use of topical MMC in this meta-analysis. Safe use of adjunct topical MMC in pediatric DCR has been described, but comparative outcomes with and without adjunct antimetabolite have not been reported (10, 51).

There have been far fewer studies evaluating 5-FU-augmented DCR. Yalaz et al. found that the mucosal ostium in patients treated with adjunct topical 5-FU during DCR resulted in decreased fibroblast activity and improved surgical outcomes (31). On the contrary, recent histopathology of topical 5-FU in rabbit models revealed increased nasolacrimal tissue inflammation and fibrosis (52). The effect of DCR outcome with topical application of 5-FU remains controversial (27–32).

Only two studies have previously evaluated intramucosal injection of antimetabolites during DCR. Costa et al. evaluated the role of intramucosal injection of 5-FU during DCR in adult patients and found no difference in symptomatic recurrence or surgical ostium size 2 months post-operatively (33). However, follow up was limited to 60 days after surgery and differences in osteotomy size may not be apparent until longer follow up. Circumostial injection of MMC during DCR has also been described with 96% functional success rate and no reported adverse events (34, 53). However, this study lacked a control group to directly compare outcomes with and without injection of MMC. Circumostial injection of 5-FU and MMC appears to be safe, though their impact on outcomes remains unclear. Successful outcomes after pediatric external DCR in our study was comparable to prior published literature of DCR in pediatric patients, ranging from 83 to 97% (10–17).

The literature on 5-FU-augmented DCR showing limited effect on outcome is in contrast to results from this study in which adjunct intramucosal 5-FU for pediatric DCR was associated with a 100% reduction of recurrence (20, 22–37, 42–45, 52). Our technique involves injection directly into the circumostial nasal and lacrimal mucosa at the time of surgery, as compared to topical 5-FU applied on a pledget as described in the literature. We believe the different delivery techniques lead to differences in tissue concentration and/or duration of effect, which can result in variable outcomes. The mucosal tissue concentration of anti-metabolites after intramucosal injection has not been evaluated; however, one study showed that after a 3-minute application of topical MMC during endoscopic DCR, MMC tissue concentration decreased 3-fold within 30 minutes and was undetectable by 24 hours (35). Of course, it is the intracellular concentration that provides the intended pharmacologic effect, and drug uptake is influenced by drug concentration. Intramucosal injection of anti-metabolites may result in higher initial tissue concentrations with prolonged duration compared to topical delivery, resulting in greater effect on fibroblasts which have a doubling time of 24 hours and explaining the difference in outcomes (34, 37). Alternatively, antimetabolites may be more efficacious in decreasing recurrence in pediatric patients due to a more robust post-operative fibrotic response in this population.

Our results revealed no difference in recurrence between patients with and without craniofacial disorders as neither group had recurrence. The 0% rate of recurrence in patients with craniofacial disorders in our study may be secondary to small sample size, surgical technique, or use of intramucosal 5-FU injection. Patients with Down syndrome or other syndromes with associated craniofacial abnormalities have a higher incidence of NLDO and 3-fold higher risk of failure after primary treatment for NLDO (54–57). Risk factors for failed probing in Down syndrome patients include bilateral NLDO, obstruction at multiple levels, and younger age at treatment (58). Higher rates of recurrence in Down syndrome are in part due to anatomical differences, including reduced nasolacrimal duct dimensions and bony or membranous obstructions other than persistent Hasner’s membrane (59, 60). Studies evaluating outcomes after DCR in pediatric patients with craniofacial syndromes are limited. Most large series of pediatric DCR exclude patients with associated craniofacial syndromes or contain only a limited number of cases.

Limitations of this study include its retrospective nature, lack of a control arm which limits the ability to claim DCR with 5-FU is superior to standard DCR, lack of data on follow-up duration, and use of data from multiple surgeons and hence slight differences in surgical techniques. Pediatric NLDO requiring intervention with DCR surgery is uncommon, and this study adds a significant number of patients to the published literature.

We advocate the use of intramucosal 5-FU in cases of pediatric DCR at higher risk of epiphora recurrence, such as those with craniofacial abnormalities and surgical comorbidities. 5-FU is sufficiently safe, and the results sufficiently compelling, to suggest that this technique may be a valuable addition to the tools of surgeons performing DCR in the pediatric population. In summary, adjuvant intramucosal circumostial 5-FU is a safe modification to DCR surgery in pediatric patients and highly effective in decreasing rate of epiphora recurrence.

## Data Availability

The datasets presented in this study can be found in online repositories. The names of the repository/repositories and accession number(s) can be found in the article/supplementary material.
